# Fingerprinting triacylglycerols and aldehydes as identity and thermal stability indicators of camellia oil through chemometric comparison with olive oil

**DOI:** 10.1002/fsn3.2209

**Published:** 2021-03-06

**Authors:** Ling Peng, Jieyao Yuan, Dan Yao, Chi Chen

**Affiliations:** ^1^ Department of Food Science and Nutrition University of Minnesota St. Paul MN USA; ^2^ Department of Food Science Yichun University Yichun China

**Keywords:** aldehyde, camellia oil, LC‐MS‐based chemometrics, olive oil, thermal stability, triacylglycerol

## Abstract

Camellia oil is widely recognized as a high‐quality culinary oil in East Asia for its organoleptic and health‐promoting properties, but its chemical composition and thermal stability have not been comprehensively defined by comparisons with other oils. In this study, the triacylglycerols (TAGs) in camellia, olive, and six other edible oils were profiled by the liquid chromatography‐mass spectrometry (LC‐MS)‐based chemometric analysis. Besides observing the similarity between camellia oil and olive oil, TAG profiling showed that OOO, POO, and OOG (O: oleic acid, P: palmitic acid, and G: gadoleic acid) can jointly serve as the identity markers of camellia oil. Thermal stability of virgin camellia oil (VCO) was further evaluated by extensive comparisons with virgin olive oil (VOO) in common lipid oxidation indicators, aldehyde production, and antioxidant and pro‐oxidant contents. The results showed that *p*‐anisidine value (AnV) was the sensitive lipid oxidation indicator, and C9‐C11 aldehydes, including nonanal, 2‐decenal, 2,4‐decadienal, and 2‐undecenal, were the most abundant aldehydes in heated VCO and VOO. Under the frying temperature, heated VCO had lower AnV and less aldehydes than heated VOO. Interestedly, the VCO had lower levels of pro‐oxidant components, including α‐linolenic acid, free fatty acids, and transition metals, as well as lower levels of antioxidants, including α‐tocopherol and phenolics, than the VOO. Overall, great similarities and subtle differences in TAG and aldehyde profiles were observed between camellia and olive oils, and the thermal stability of camellia oil might be more dependent on the balance among its unsaturation level, pro‐oxidant, and antioxidant components than a single factor.

## Graphical abstract

OOO, POO, and OOG are a cluster of triacylglycerols distinguishing camellia oil from other oils. *p*‐Anisidine value and C9‐C11 aldehydes are robust indicators of oxidative status for camellia and olive oils. Among them, nonanal, 2‐decenal, 2,4‐decadienal, and 2‐undecenal are the most abundant aldehydes in both oils. Instead of a single factor, the thermal stability of camellia and olive oils is more dependent on the balance among fatty acid unsaturation level, pro‐oxidant, and antioxidant components.

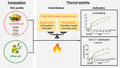

## 1 INTRODUCTION

*Camellia oleifera*, commonly known as the tea oil camellia, is widely cultivated in East Asia for producing camellia oil, or tea seed oil (Yang et al., 2016). Camellia oil is generally considered as a high‐quality culinary oil for its organoleptic properties in color and flavor, high smoke point, and health‐promoting phytochemical contents including tocopherols, catechins, saponins, and squalenes (Chou et al., 2018; Lee & Yen, 2006; Lee et al., 2018; Shi et al., 2020). Enriched with more than 70% oleic acid, a monounsaturated fatty acid (MUFA), camellia oil is more suitable for frying and other high‐temperature processing than the oils rich in polyunsaturated fatty acids (PUFAs), such as soybean, corn, and flaxseed oils, which are high in linoleic acid or α‐linolenic acid. In practice, camellia oil has been blended with these oils to improve their stability and performance in the frying operations of fast food industry (Li et al., 2014; Wang, Sui, et al., 2016).

Current knowledge on the chemical properties that distinguish camellia oil from other culinary oils in chemical composition and thermal stability stays limited. For example, the fatty acid composition of camellia oil is known to be similar to that of olive oil (Cao et al., 2020; Liu et al., 2017). However, in edible oils, fatty acids mainly exist in esterified forms. Therefore, the triacylglycerol (TAG) profile is more relevant to the chemical and physical properties of an edible oil than its fatty acid profile. Although the TAG profile of camellia oil has been examined previously (Wei et al., 2016; Zeb, 2012), a comprehensive comparison of camellia oil with other edible oils especially olive oil in their TAG profiles has been rarely conducted. In addition, as a frying oil, camellia oil is expected to undergo the thermal stress‐elicited changes through hydrolysis, oxidation, and polymerization reactions, forming reactive and potentially toxic lipid oxidation products (LOPs) (Choe & Min, 2007), but the kinetics of forming LOPs, especially the profile of diverse aldehydes, in heated camellia oil was also not well examined.

The paucity of comprehensive comparisons on the TAG and LOP profiles of camellia oil with other oils, especially olive oil, could be partially attributed to the limited coverage of traditional analytical platforms, which only target predefined compounds or properties, such as fatty acids, thiobarbituric acid reactive substances (TBARS), or specific aldehydes. In this study, both targeted chemical analyses and untargeted chemometric analyses combining chemical derivatization, high‐resolution LC‐MS analysis, and multivariate modeling were utilized to define the chemical characteristics that differentiate camellia oil from other common plant‐derived edible oils, especially olive oil, in chemical composition and thermal stability.

## 2 MATERIALS AND METHODS

### 2.1 Vegetable oils and chemicals

Virgin camellia oil (VCO), virgin olive oil (VOO), refined olive oil (ROO), canola oil (CNO), corn oil (COO), grapeseed oil (GPO), peanut oil (PNO), safflower oil (SFO), and soybean oil (SBO) were purchased from local grocery stores. Refined camellia oil (RCO) was obtained from Jiangxi Xing‐Huo Biotechnology. Except for camellia and olive oils, other tested oils were not specifically labeled as virgin or refined. The sources of the chemicals and reagents used in chemical analysis, LC‐MS analysis, structural confirmation, and quantification were included in the supplementary data (Table S1).

### 2.2 Preparation of heated oils

To test their thermal stability, 300 ml of VCO and VOO were heated in a 500‐ml round‐bottom glass flask, respectively, using an electric heating mantle with power controller. The temperatures of two oils were gradually increased from room temperature (22°C) to frying temperature (185°C) for one and half hours with bubbling air (50 ml/min) and then held constantly at 185°C for 6 hr. The oil samples were collected at 45, 65, 85, 105, 125, 145, and 165°C, and then at 5, 15, 30 min, 1, 2, 4, and 6 hr of 185°C (Figure S2a). All samples were stored at −80°C prior to further analysis.

### 2.3 Measurements of lipid oxidation indicators

The peroxide values (PVs) and TBARS values of oil samples were measured in triplicate using methods previously reported in this laboratory (Wang, Csallany, et al., 2016). The *p‐*anisidine value (AnVs) of oil samples were measured in triplicate following the American Oil Chemists’ Society (AOCS) method Cd 18‐90 (AOCS, 2017).

### 2.4 LC‐MS analysis of TAG

Neutral lipids, mainly acylglycerols, in edible oils were detected by the LC‐MS analysis in the forms of their ammonium adducts (Wang, Yao, et al., 2018). All the oil samples (in triplicate) were first diluted 100,000 times with n‐butanol containing 1 μg/ml tripentadecanoin as the internal standard, and then transferred to LC vials. For LC‐MS analysis, 5 µl of diluted oil sample was injected into an ultra‐performance liquid chromatography (UPLC) system (Waters) and separated by a BEH C8 2.1 × 50 mm, 1.7 μm particle size column (Waters) using a mobile phase gradient ranging from 55% of mobile phase A (water: acetonitrile = 60:40, v/v, containing 10 mmol/L ammonium formate and 0.1% formic acid) to 100% of mobile phase B (methanol containing 10 mmol/L ammonium formate and 0.1% formic acid) at 60°C over a 10 min run. The LC eluent was introduced into a Xevo‐G2‐S quadrupole time‐of‐flight mass spectrometer (QTOFMS, Waters) for ionization and MS scan. TAG ammonium adducts were detected at positive mode with 3 kV and 30 V of capillary voltage and cone voltage, respectively, for electrospray ionization. Source temperature and desolvation temperature were set at 120 and 350°C, respectively. Nitrogen was used as the cone gas (50 L/hr) and the desolvation gas (600 L/hr) while argon as the collision gas. Accurate mass measurement was achieved by the calibration using sodium formate solution (ranging *m/z* 50–1,500) and the intermittent injection of lock mass leucine enkephalin ([M + H]^+^ = *m/z* 556.2771) in every run. The structure identification of TAG species was determined by accurate mass measurement, elemental composition analysis, database search, tandem mass spectrometry (MS/MS) fragmentation with collision energies ranging from 10 to 60 eV, and comparisons with authentic standards if available. The levels of individual TAGs were determined by their relative abundances.

### 2.5 LC‐MS analysis of aldehydes in oil samples

Prior to the LC‐MS analysis, aldehydes in oil samples were derivatized by 2‐hydrazinoquinoline (HQ) based on a previously established method (Wang, Csallany, et al., 2016). Briefly, 2 µl of oil sample (in triplicate) was added into 70 µl of freshly prepared acetonitrile solution including 1 mmol/L 2,2′‐dipyridyl disulfide (DPDS), 1 mmol/L triphenylphosphine (TPP), 1 mmol/L HQ, and 100 µmol/L acetone‐d6 (internal standard). The mixture was incubated at 60°C for 30 min, and the reaction was terminated by chilling samples on ice and adding 100 µl of water. After vortexing and centrifuging at 21,000× *g* for 10 min, the supernatant was transferred into an LC vial for LC‐MS analysis. The sample was separated by a UPLC system (Waters) equipped with a BEH C18 2.1 × 50 mm, 1.7 μm particle size column (Waters) using a mobile phase gradient (A: H_2_O containing 0.05% acetic acid, v/v, and 2 mmol/L ammonium acetate; B: H_2_O/acetonitrile = 5:95, v/v, containing 0.05% acetic acid, v/v, and 2 mmol/L ammonium acetate) for separation. The conditions of MS analysis and the methods for structure confirmation were the same as the TAG analysis. Quantitative analysis was conducted by peak integration and standard curve using QuanLynx™ software (Waters).

### 2.6 Chemometric modeling and data visualization of TAG and aldehyde profiles

Mass chromatograms and mass spectral data were acquired and processed by MarkerLynx™ software (Waters) to generate a multivariate data matrix, which was subsequently exported into the SIMCA‐P+™software (Umetrics) and transformed by Pareto scaling. To establish a model for the data matrix and define the correlations among samples, unsupervised principal component analysis (PCA) or supervised orthogonal partial least squares‐discriminant analysis (OPLS‐DA) was performed. The chemical markers contributing to the sample separation were identified in the loadings plot of the model. The correlations among these identified markers were defined by hierarchical cluster analysis (HCA) and heat maps generated by the R program (http://www.R‐project.org) based on their relative abundance after *Z* score transformation.

### 2.7 LC‐MS analysis of total and free fatty acids

The composition of total and free fatty acids in oil samples was measured in triplicate by a previously established LC‐MS method (Yuan et al., 2020). To determine the composition of total fatty acids, oil samples were first treated by alkaline hydrolysis to release the fatty acids from TAGs. Briefly, 5 μl of 5% oil dissolved in n‐butanol was mixed with 200 μl of methanol containing 200 μmol/L of labeled ^13^C_2_‐palmitic acid as the internal standard and 35 μl of 40% potassium hydroxide (w/v), and then incubated at 60°C for 30 min. After cooling, the mixture was neutralized by 80 μl of 2.5 mol/L HCl and 200 μl of 200 mmol/L phosphate buffer (pH = 7), and then centrifuged at 18,000× *g* for 10 min. The supernatant underwent HQ derivatization and LC‐MS analysis following the same procedure of aldehyde analysis. As for the composition of free fatty acids, oil samples were derivatized directly without hydrolysis prior to the LC‐MS analysis. The composition of total fatty acids was reported as the percentages of individual fatty acids in total fatty acids while the composition of free fatty acids was reported as the concentrations of individual fatty acids in oil samples.

### 2.8 Antioxidant analysis

#### 2.8.1 Trolox equivalent antioxidant capacity (TEAC)

The total antioxidant capacity of oil samples was measured in triplicate based on a radical quenching method (Pellegrini et al., 2001). The TEAC value was calculated using a trolox standard curve ranging from 0 to 15 μmol/L.

#### 2.8.2 Total phenolic content

Phenolics were first extracted from the oils in triplicates (Wang et al., 2017), and their levels were determined using the Folin–Ciocalteu method (Gutfinger, 1981). Caffeic acid, as a reference compound, was diluted in 60% aqueous methanol for standard curve preparation.

#### 2.8.3 α‐Tocopherol

α‐Tocopherol in oil samples was extracted in triplicate through an ultrasound‐assisted saponification method as described by Zhang, Wang, et al. (2019). Concentrations of α‐tocopherol were quantified using its standard and the same LC‐MS method for TAG analysis.

### 2.9 Iron and copper contents

Iron and copper in oil samples were measured in triplicate using a modified atomic absorption spectrometry method (Zhong et al., 2016). Briefly, 0.25 g of oil sample was added into 5 ml of trace metal grade nitric acid (67%–70%) and digested in a microwave digestion system (Discover SP‐D digester, CEM Corporation) using a program with a temperature gradient of 5 min from room temperature to 200°C and 5 min at 200°C. The maximum pressure and power were set at 400 psi and 300 W, respectively. The digested solution was diluted with 0.2% nitric acid in a 5‐ml volumetric flask, and then, a 20 µl aliquot was injected together with the modifier (magnesium nitrate) to a graphite furnace atomic absorption spectrometry (GFAAS) system (AAnalyst 800, PerkinElmer). Concentrations of iron and copper were determined using their respective standard curves.

### 2.10 Statistical analysis

Statistical analysis was conducted by the GraphPad Prism 8.0 software (GraphPad Software). Data were expressed as the mean ± standard deviation (*SD*) from three measurements. The statistical differences among 10 vegetable oils were determined by one‐way ANOVA and Tukey post hoc test and the statistical differences between VCO and VOO by the two‐tailed Student's *t* test, with *p* < .05 as statistically significant.

## 3 RESULTS

### 3.1 Chemometric comparison of TAG profiles between camellia oil and common plant‐derived edible oils

**FIGURE 1:**
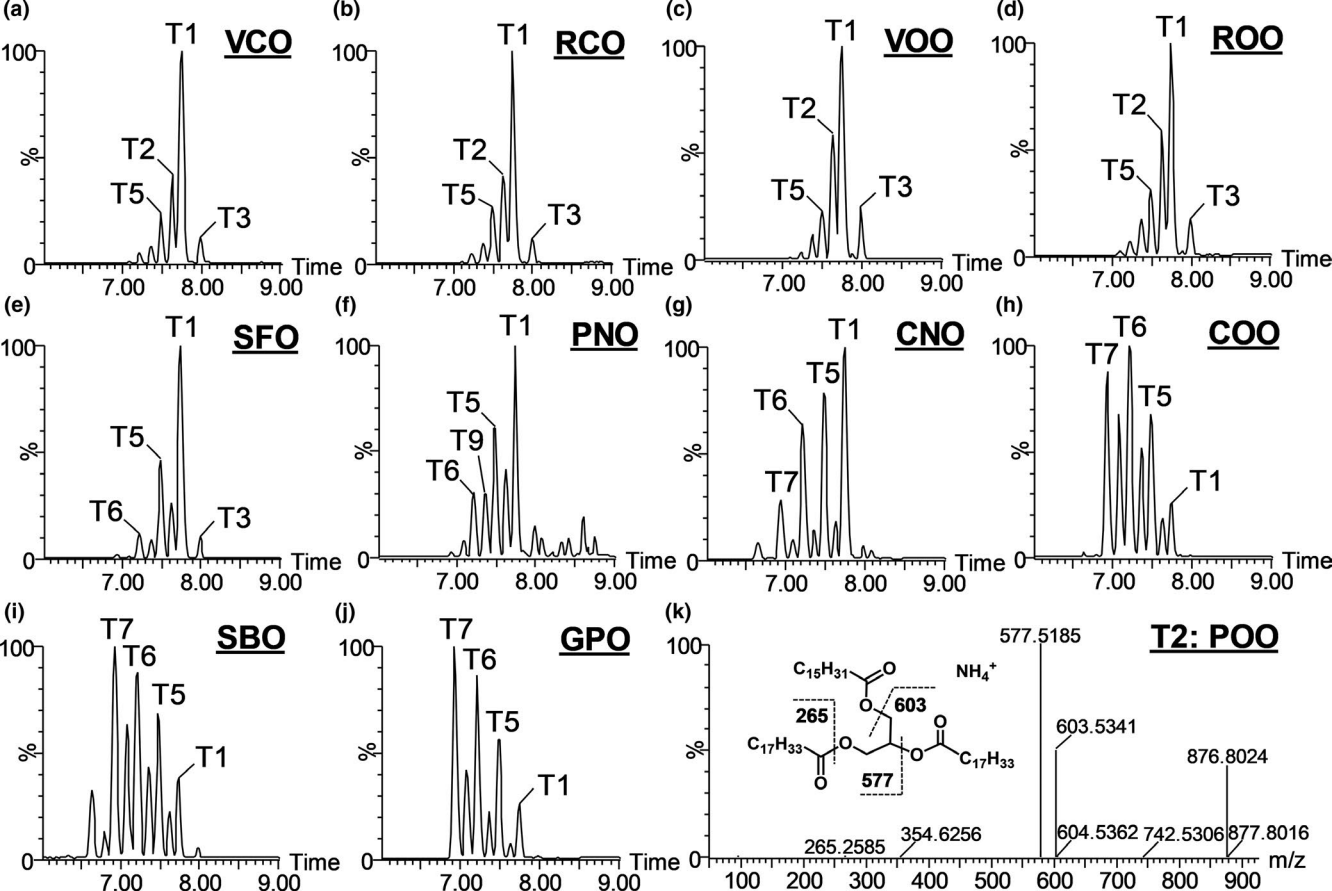
Triacylglycerols (TAGs) in 10 vegetable oils. (a–j) Representative LC chromatograms (6–9 min) of TAGs in 10 vegetable oils. VCO, virgin camellia oil; RCO, refined camellia oil; VOO, virgin olive oil; ROO, refined olive oil; SFO, safflower oil; PNO, peanut oil; CNO, canola oil; COO, corn oil; SBO, soybean oil; GPO, grapeseed oil. Major peaks are labeled, and their ID are listed in Table 1, including T1 (OOO), T2 (POO), T3 (SOO), T5 (OOL), T6 (OLL), T7 (LLL), and T9 (POL). P: palmitic acid; S: stearic acid; O: oleic acid; L: linoleic acid. (k) Representative TAG fragmentogram of T2 (POO). P: palmitic acid; O: oleic acid

**FIGURE 2:**
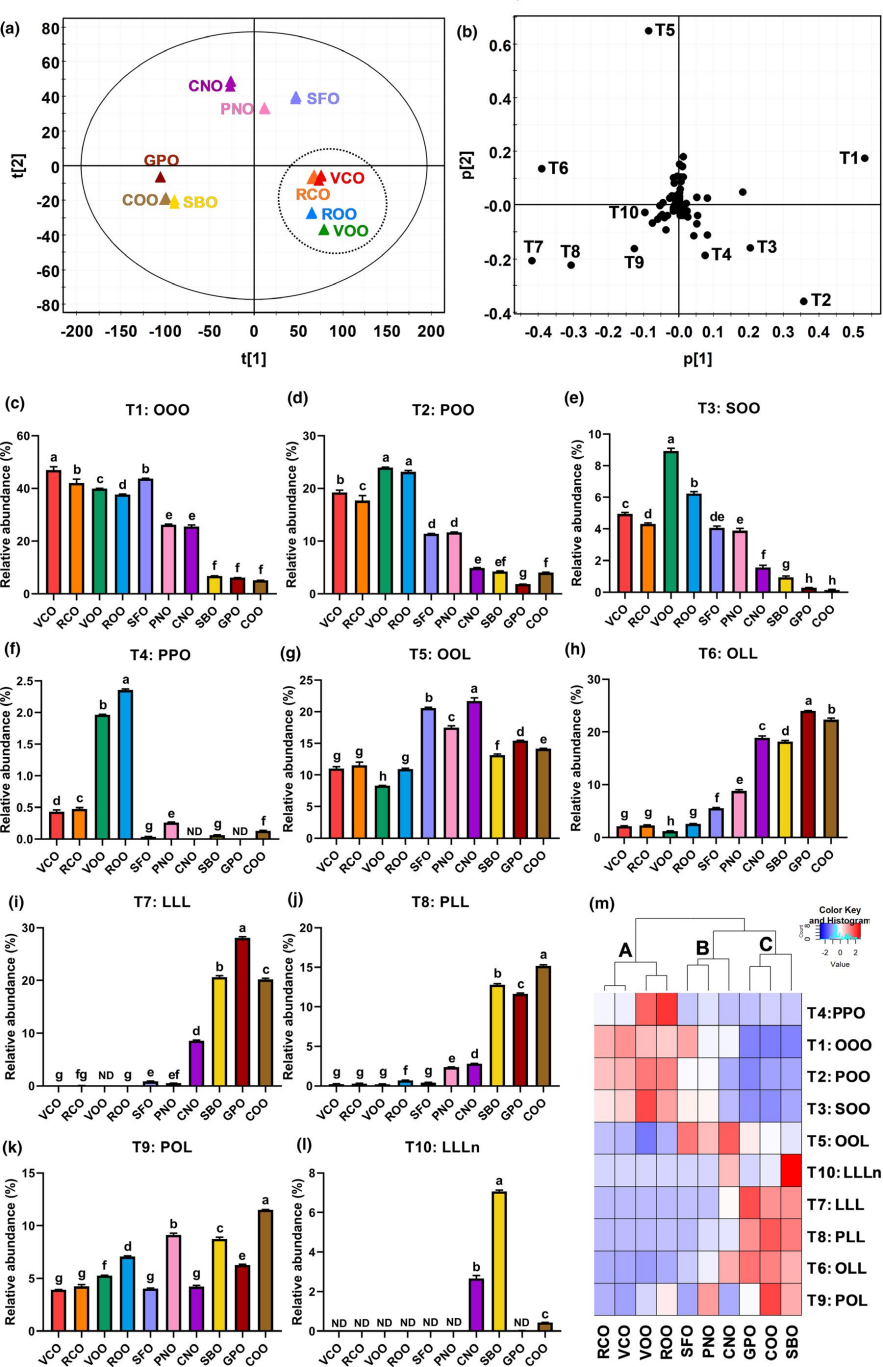
Chemometric analysis of 10 vegetable oils based on their triacylglycerol (TAG) composition. Each oil was analyzed in triplicate. (a) The scores plot of the principal component analysis (PCA) model. The t[1] and t[2] values are the scores of each sample in the principal components 1 and 2, respectively. (b) The loadings plot of the PCA model. Major TAG markers (T1–T10) contributing to sample separation are labeled. The p[1] and p[2] values are the contributing weights of each ion to the principal components 1 and 2 of the PCA model, respectively. (c‐l) Distribution of TAG markers in 10 vegetable oils, including (c) T1 (OOO), (d) T2 (POO), (e) T3 (SOO), (f) T4 (PPO), (g) T5 (OOL), (h) T6 (OLL), (i) T7 (LLL), (j) T8 (PLL), (k) T9 (POL), and (l) T10 (LLLn). O: oleic acid; P: palmitic acid; S: stearic acid; L: linoleic acid; Ln: linolenic acid. *p* < .05 is considered as statistically significant. Different letters represent the significant differences among oils samples. ND means not detected. (m) Heat map and dendrogram of TAGs from the cluster analysis on 10 oils. The TAG markers are clustered in three groups: clusters A, B, and C

**FIGURE 3:**
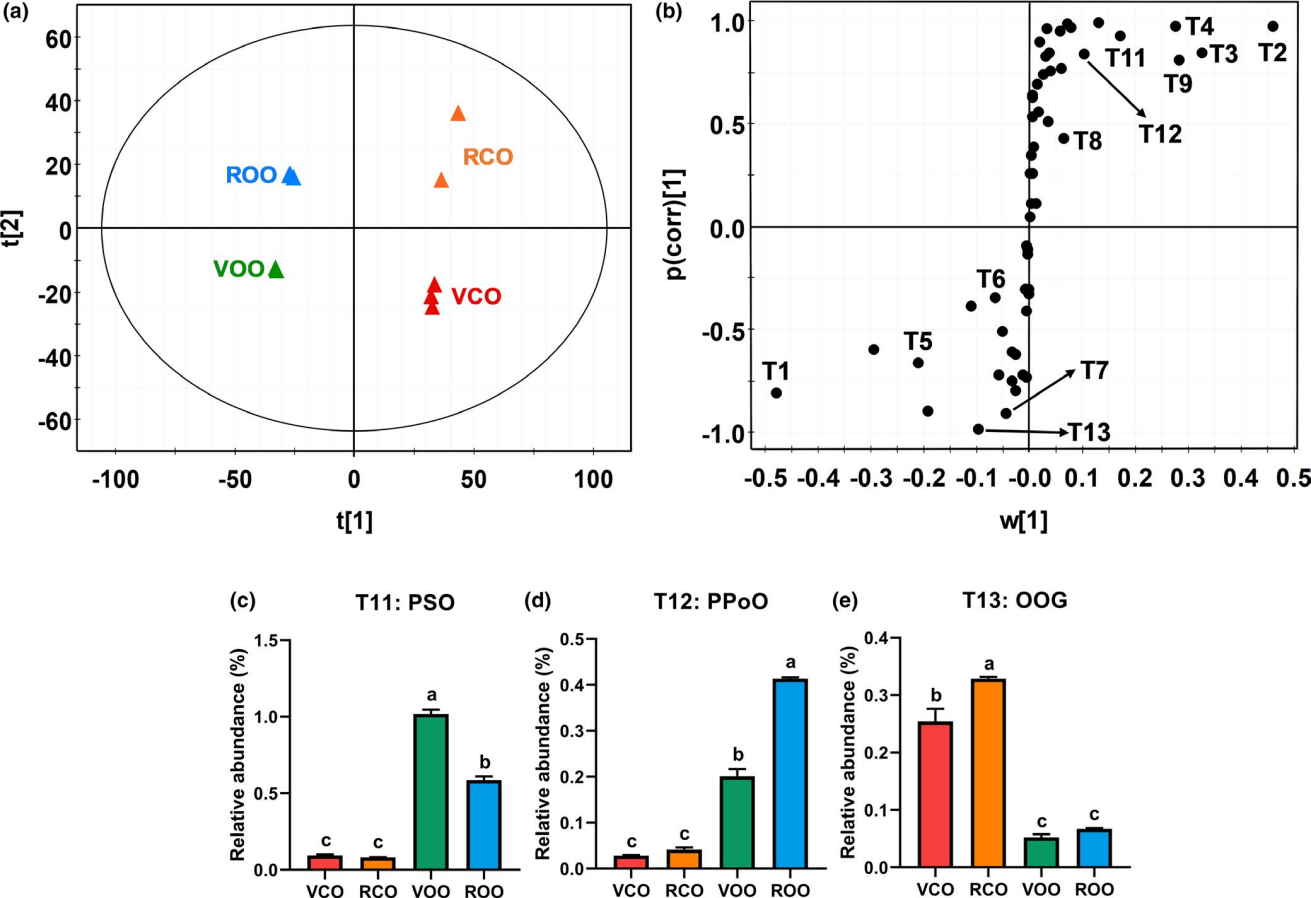
Chemometric analysis of camellia oils (VCO and RCO) and olive oils (VOO and ROO) based on their triacylglycerol (TAG) profiles. Each oil was analyzed in triplicate. VCO: virgin camellia oil; RCO: refined camellia oil; VOO: virgin olive oil; ROO: refined olive oil. (a) The scores plot of a principal component analysis (PCA) model on VCO, RCO, VOO, and ROO. (b) The S‐loadings plot of an orthogonal partial least squares‐discriminant analysis (OPLS‐DA) model distinguishing camellia oil (VCO and RCO) from olive oil (VOO and ROO). Major TAG markers (T1–T9 and T11–T13) contributing to the separation are labeled. (c‐e) Relatively abundancies of TAG markers, including (c) T11 (PSO), (d) T12 (PPoO), and (e) T13 (OOG). P: palmitic acid; S: stearic acid; O: oleic acid; Po: palmitoleic acid. *p* < .05 is considered as statistically significant. Different letters indicate the significant differences among oil samples

TAGs in VCO and RCO were compared with eight different plant‐derived edible oils, including SFO, PNO, CNO, COO, SBO, GPO as well as VOO and ROO, through the LC‐MS‐based chemometric analysis. As shown in the representative chromatograms, the distribution of major TAGs differed greatly among examined oils (Figure 1a–j). The fatty acid composition of these TAGs was determined by the MS/MS fragmentation that generates the diacylglycerol and monoacylglycerol fragments from the neutral loss of acyl groups (Table 1). For example, POO (T2, *m/z* = 876.8024) had two major fragments (*m/z* = 577.5185 and *m/z* = 603.5341) from the neutral losses of oleoyl group and palmitoyl group, respectively, and one minor fragment (*m/z* = 265.2585) belonging to the oleic acid (Figures 1k and S1). In the scores plot of the PCA model on the TAG profiles of these oils, VCO and RCO were close to VOO and ROO while separated from GPO, COO, SBO, SFO, PNO, and CNO (Figure 2a). This distribution pattern suggested that the TAG profiles of camellia oils were comparable to those of olive oils but different from other vegetable oils. Major TAGs (T1–T10) contributing to the sample separation in the PCA model were identified in the loadings plot (Figure 2b). Their relative abundances were compared across examined oils (Figure 2c–l). The distribution pattern of identified TAG markers in all examined oils was further examined by the HCA and visualized by a heat map, in which three major clusters (labeled as A, B, and C) were observed (Figure 2m). RCO, VCO, VOO, and ROO were grouped in cluster A due to the high abundances of oleic acid‐containing TAGs, including T1 (OOO), T2 (POO), T3 (SOO), and T4 (PPO). SFO, PNO, and CNO in cluster B had the high intensity of T5 (OOL), while GPO, COO, and SBO in cluster C were particularly abundant in linoleic acids, such as T7 (LLL), T8 (PLL), T6 (OLL), and T9 (POL). An additional PCA modeling was conducted to specifically compare the TAG profiles of camellia oil and olive oil. The scores plot showed that VCO and RCO can be separated from VOO and ROO along with the 1st principal component (PC) of the model (Figure 3a). The TAGs contributing to this separation (T1–T9 and T11–T13) were further identified in the S‐plot of an OPLS‐DA model (Table 1; Figure 3b), including T4 (PPO), T11 (PSO), T12 (PPoO), and T13 (OOG), which are 4 minor TAGs with more than four fold differences between camellia oil and olive oil (Figures 2f and 3c–e). T4, T11, and T12, which are more abundant in olive oil, contain palmitic acid, while T13, which is more abundant in camellia oil, contains gadoleic acid (Table 1).

**TABLE 1:** Major TAG species contributing to the classification of 10 vegetable oils in the PCA model and clustering analysis

ID	Formula	TAG composition^a^	Calculated exact mass of [M + NH_4_]^+^	Measured mass of [M + NH_4_]^+^	Mass deviation (ppm)	Major fragments of MS/MS (*m/z*)
T1	C_57_H_104_O_6_	OOO	902.8177	902.8175	−0.22	603
T2	C_55_H_102_O_6_	POO	876.8020	876.8022	0.23	577, 603
T3	C_57_H_106_O_6_	SOO	904.8333	904.8338	0.55	603, 605
T4	C_53_H_100_O_6_	PPO	850.7864	850.7868	0.47	551, 577
T5	C_57_H_102_O_6_	OOL	900.8020	900.8022	0.22	601, 603
T6	C_57_H_100_O_6_	OLL	898.7864	898.7868	0.45	599, 601
T7	C_57_H_98_O_6_	LLL	896.7707	896.7694	−1.45	599
T8	C_55_H_98_O_6_	PLL	872.7707	872.7698	−1.03	575, 599
T9	C_55_H_100_O_6_	POL	874.7864	874.7866	0.23	575, 577, 601
T10	C_57_H_96_O_6_	LLLn	894.7551	894.7545	−0.67	597, 599
T11	C_55_H_104_O_6_	PSO	878.8177	878.8181	0.46	577, 579, 605
T12	C_53_H_98_O_6_	PPoO	848.7707	848.7714	0.82	549, 575, 577
T13	C_59_H_108_O_6_	OOG	930.8490	930.8497	0.75	603, 631

Abbreviations: G, gadoleic acid; L, linoleic acid; Ln, linolenic acid; O, oleic acid; P, palmitic acid; PCA, principal component analysis; Po, palmitoleic acid; S, stearic acid; TAG, triacylglycerol.

^a^ The stereospecific numbering (*sn*) positions of three fatty acids in the glycerol backbone of TAGs were not determined.

### 3.2 Fatty acid composition of VCO and VOO

To confirm the observed differences in the TAG profiles of camellia oil and olive oil, the fatty acid compositions of VCO and VOO were determined. Both VCO and VOO were dominated by oleic acid (C18:1), followed by linoleic acid (C18:2), palmitic acid (C16:0), and stearic acid (C18:0) as the major fatty acids (Table 2). A prominent difference between the two oils was on the saturation level. Almost all saturated fatty acids (SFAs) in VOO, including major ones (palmitic acid and stearic acid) and minor ones (lauric acid, myristic acid, and arachidic acid), were present in greater abundances than those in VCO. Reciprocally, VCO had a higher unsaturation level than VOO. However, within MUFAs, palmitoleic acid (C16:1) was more abundant in VOO while gadoleic acid (C20:1) was more abundant in VCO (Table 2).

**TABLE 2:** Fatty acid composition of VCO and VOO

Fatty acid composition (%)	VCO	VOO	Significance^a^
Lauric acid (C12:0)	0.014 ± 0.004	0.039 ± 0.008	*
Myristic acid (C14:0)	0.014 ± 0.005	0.034 ± 0.005	*
Palmitic acid (C16:0)	7.380 ± 0.709	11.025 ± 2.536	—
Palmitoleic acid (C16:1)	0.008 ± 0.001	0.289 ± 0.060	*
Heptadecanoic acid (C17:0)	0.022 ± 0.005	0.018 ± 0.005	—
Heptadecenoic acid (C17:1)	0.004 ± 0.000	0.005 ± 0.000	—
Stearic acid (C18:0)	1.999 ± 0.420	4.201 ± 0.072	*
Oleic acid (C18:1)	79.851 ± 2.097	73.363 ± 0.977	*
Linoleic acid (C18:2)	10.020 ± 0.867	7.294 ± 1.408	‐
α‐Linolenic acid (C18:3)	0.298 ± 0.047	0.717 ± 0.131	*
Arachidic acid (C20:0)	0.022 ± 0.005	0.613 ± 0.104	*
Gadoleic acid (C20:1)	0.370 ± 0.056	0.077 ± 0.020	*
Σ SFAs	9.451 ± 1.145	15.930 ± 2.638	*
Σ MUFAs	80.233 ± 2.048	73.734 ± 0.922	*
Σ PUFAs	10.318 ± 0.914	8.011 ± 1.524	—
Σ UFAs	90.552 ± 1.150	81.744 ± 1.522	*

Note

Results were expressed as means ± standard deviation of triplicates.

Abbreviations: PCA, principal component analysis; VCO, virgin camellia oil; VOO, virgin olive oil.

^a^ *p* < 0.05 is considered as statistically significant. *: significance; —: nonsignificance.

### 3.3 Thermal stability evaluation by conventional lipid oxidation indicators

Because of their similarities in TAGs and fatty acids, VCO and VOO were compared on the chemical changes occurred under thermal stress. At the frying temperature (Figure S2a), VCO and VOO underwent gradual and visible changes in color (Figure S2b–c). The stability of VCO and VOO was first evaluated by measuring PV, TBARS, and AnV, three common indicators of lipid oxidation.

**FIGURE 4:**
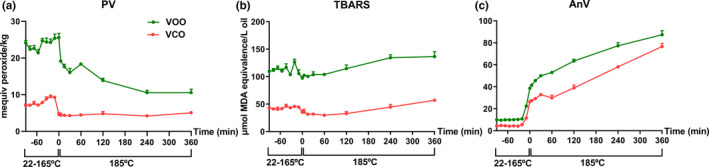
Profiles of common lipid oxidation indicators in control and heated virgin camellia oil (VCO) and virgin olive oil (VOO). Each oil sample was analyzed in triplicate. (a) Peroxide value (PV). (b) Thiobarbituric acid reactive substances (TBARS) value. (c) *p*‐Anisidine value (AnV)

PV: The basal PV of VOO before heating was greater than that of VCO. After heating, the PVs of VCO and VOO were peaked when the temperature reached 165 and 185°C, respectively, and then decreased rapidly at 185°C (Figure 4a).

TBARS: Similar to PV, the basal TBARS value of VOO was greater than that of VCO. However, the TBARS values of VOO and VCO were not greatly affected by the thermal treatment (Figure 4b).

AnV: The basal AnV of VOO was also greater than that of VCO. Compared with PV and TBARS, AnV was far more sensitive to the thermal treatment since it increased rapidly from 145 to 185°C and then continuously increasing at 185°C in both VCO and VOO (Figure 4c). The increase of AnV in VOO was greater than that in VCO.

### 3.4 Thermal stability evaluation by aldehyde profile

**FIGURE 5:**
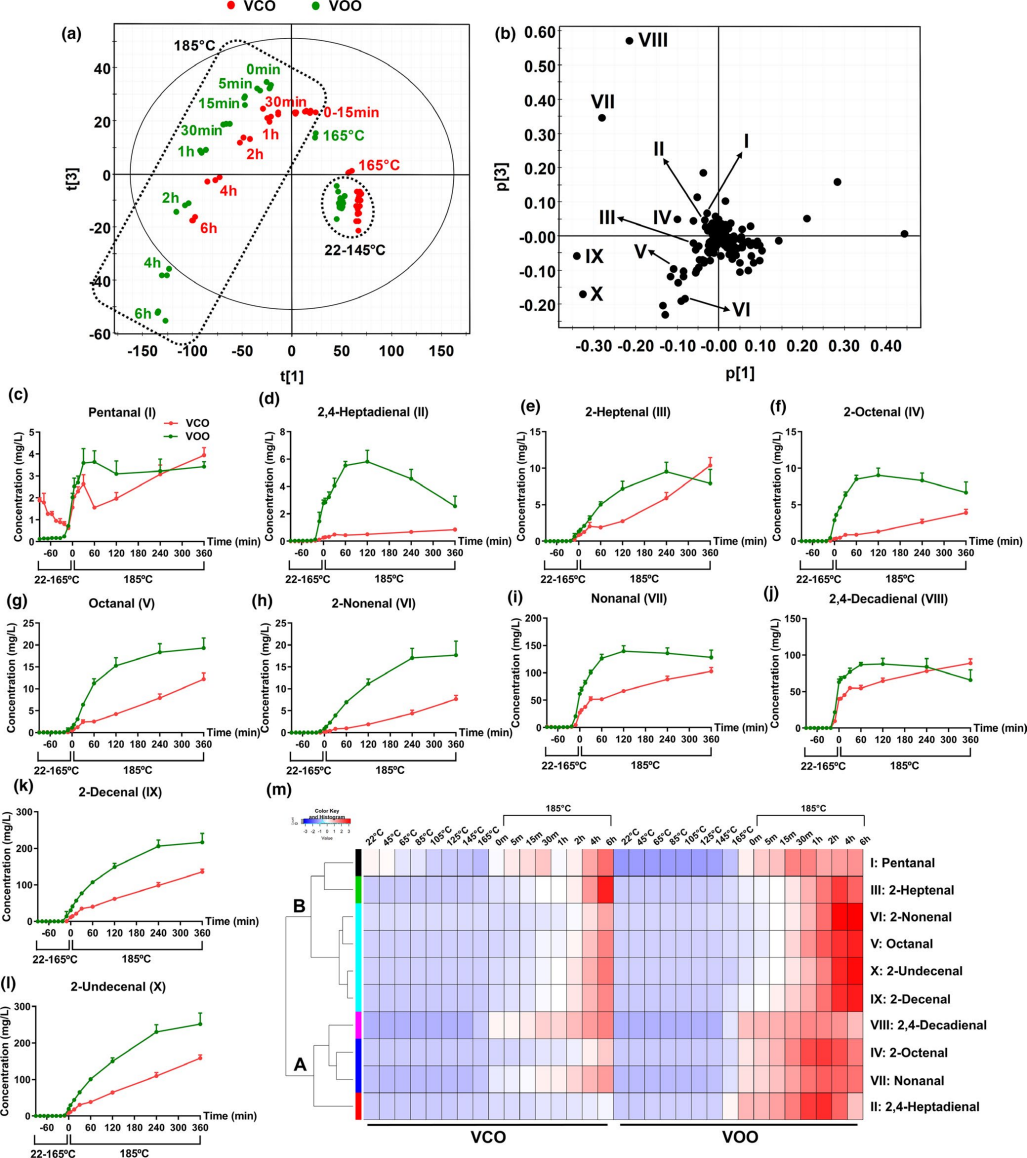
Chemometric analysis of aldehydes in control and heated virgin camellia oil (VCO) and virgin olive oil (VOO). Each oil sample was analyzed in triplicate. (a) The scores plot of a principal component analysis (PCA) model on heated VCO and VOO. (b) The loadings plot of the PCA model. Major aldehyde markers (I‐X) contributing to sample separation are labeled and quantified in Figure 5c–l. (c–l) Profiles of major aldehyde markers, including (c) Pentanal (I), (d) 2,4‐Heptadienal (II), (e) 2‐Heptenal (III), (f) 2‐Octenal (IV), (g) Octanal (V), (h) 2‐Nonenal (VI), (i) Nonanal (VII), (j) 2,4‐Decadienal (VIII), (k) 2‐Decenal (IX), and (l) 2‐Undecenal (X). (m) Heat map and dendrogram from the clustering analysis on aldehydes, which are clustered in two groups: cluster A and B

The increase in AnV, an index of reactive aldehydes, in both VCO and VOO after heating suggests that the aldehydes from lipid oxidation could be sensitive indicators of thermal stability for both camellia oil and olive oil. Hence, an LC‐MS‐based chemometric analysis was conducted to profile the formation and kinetics of individual aldehydes. The distribution pattern of VCO and VOO in the scores plot of a PCA model revealed three prominent features of heating‐induced aldehyde formation (Figure 5a): (a) VCO and VOO samples collected from 22 to 145°C were clustered, indicating that VCO and VOO were rather resistant to the degradation to aldehydes within this temperature range; (b) above 145°C, the clear shifts of VCO and VOO were observed in the plot, indicating dramatic increases of aldehydes in VOO and VCO; (c) the trajectories of VCO and VOO samples during 6 hr of heating at 185°C showed that both oils underwent gradual changes in their aldehyde profiles at the frying temperature, but the scale of change in VOO was greater than that in VCO, making the aldehyde profile of VOO at 2 hr of 185°C heating comparable to that of VCO at 6 hr of 185°C heating (Figure 5a). Major aldehydes responsible for the sample separation in the PCA model were first identified in the loadings plot as pentanal (I), 2,4‐heptadienal (II), 2‐heptenal (III), 2‐octenal (IV), octanal (V), 2‐nonenal (VI), nonanal (VII), 2,4‐decadienal (VIII), 2‐decenal (IX), and 2‐undecenal (X) (Table 3; Figure 5b), and then quantified (Figure 5c–l). C9‐C11 aldehydes, including nonanal (VII), 2,4‐decadienal (VIII), 2‐decenal (IX), and 2‐undecenal (X), were more abundant than C5‐C8 aldehydes, including pentanal (I), 2,4‐heptadienal (II), 2‐heptenal (III), 2‐octenal (IV), and octanal (V), in both heated VCO and VOO (Figure 5c–g,i–l). The kinetic profiles of aldehyde formation in heated VCO and VOO were compared by HCA, and two major clusters (A and B) of aldehydes were observed in the heat map (Figure 5m). The aldehydes in clusters A, including 2,4‐decadienal (VIII), 2‐octenal (IV), nonanal (VII), and 2,4‐heptadienal (II), had their concentrations peaked at either 1 hr or 2 hr of 185°C heating in VOO, but not in VCO (Figure 5d,f,i–j). In contrast, the aldehydes in cluster B, including pentanal (I), 2‐heptenal (III), 2‐nonenal (VI), octanal (V), 2‐undecenal (X), and 2‐decenal (IX), had more persistent and progressive increases in both VCO and VOO, especially during the 6‐hr heating at 185°C (Figure 5c,e,g–h,k–l). Interestingly, pentanal (I) was already detectable in VCO before the thermal treatment and its concentration in VCO decreased prior to 165°C (Figure 5c).

**TABLE 3:** Major aldehydes contributing to the separation of heated VCO and VOO samples in the PCA model and clustering analysis

ID	Compounds	Formula	Derivative formula	Calculated exact mass of [M + H]^+^	Measured mass of [M + H]^+^	Mass deviation (ppm)
I	Pentanal	C_5_H_10_O	C_14_H_18_N_3_^+^	228.1501	228.1498	−1.31
II	2,4‐Heptadienal	C_7_H_10_O	C_16_H_18_N_3_^+^	252.1501	252.1495	−2.38
III	2‐Heptenal	C_7_H_12_O	C_16_H_20_N_3_^+^	254.1657	254.1651	−2.36
IV	2‐Octenal	C_8_H_14_O	C_17_H_22_N_3_^+^	268.1814	268.1806	−2.98
V	Octanal	C_8_H_16_O	C_17_H_24_N_3_^+^	270.1970	270.1965	−1.85
VI	2‐Nonenal	C_9_H_16_O	C_18_H_24_N_3_^+^	282.1970	282.1966	−1.42
VII	Nonanal	C_9_H_18_O	C_18_H_26_N_3_^+^	284.2127	284.2120	−2.46
VIII	2,4‐Decadienal	C_10_H_16_O	C_19_H_24_N_3_^+^	294.1970	294.1965	−1.70
IX	2‐Decenal	C_10_H_18_O	C_19_H_26_N_3_^+^	296.2127	296.2120	−2.36
X	2‐Undecenal	C_11_H_20_O	C_20_H_28_N_3_^+^	310.2283	310.2276	−2.26

Note:

Aldehydes were detected by 2‐hydrazinoquinoline (HQ) derivatization and LC‐MS analysis. Structural confirmation was based on accurate mass measurement (mass deviation within 5 ppm of exact mass) and authentic standards.

Abbreviations: PCA, principal component analysis; VCO, virgin camellia oil; VOO, virgin olive oil.

### 3.5 Antioxidant and pro‐oxidant contributors to thermal stability of VCO and VOO

To further understand the chemical components contributing to the thermal stability of examined VCO and VOO, their antioxidant components were evaluated by analyzing TEAC, total phenolic content, and α‐tocopherol, while their pro‐oxidant components were evaluated by analyzing their mineral and free fatty acid contents.

**FIGURE 6:**
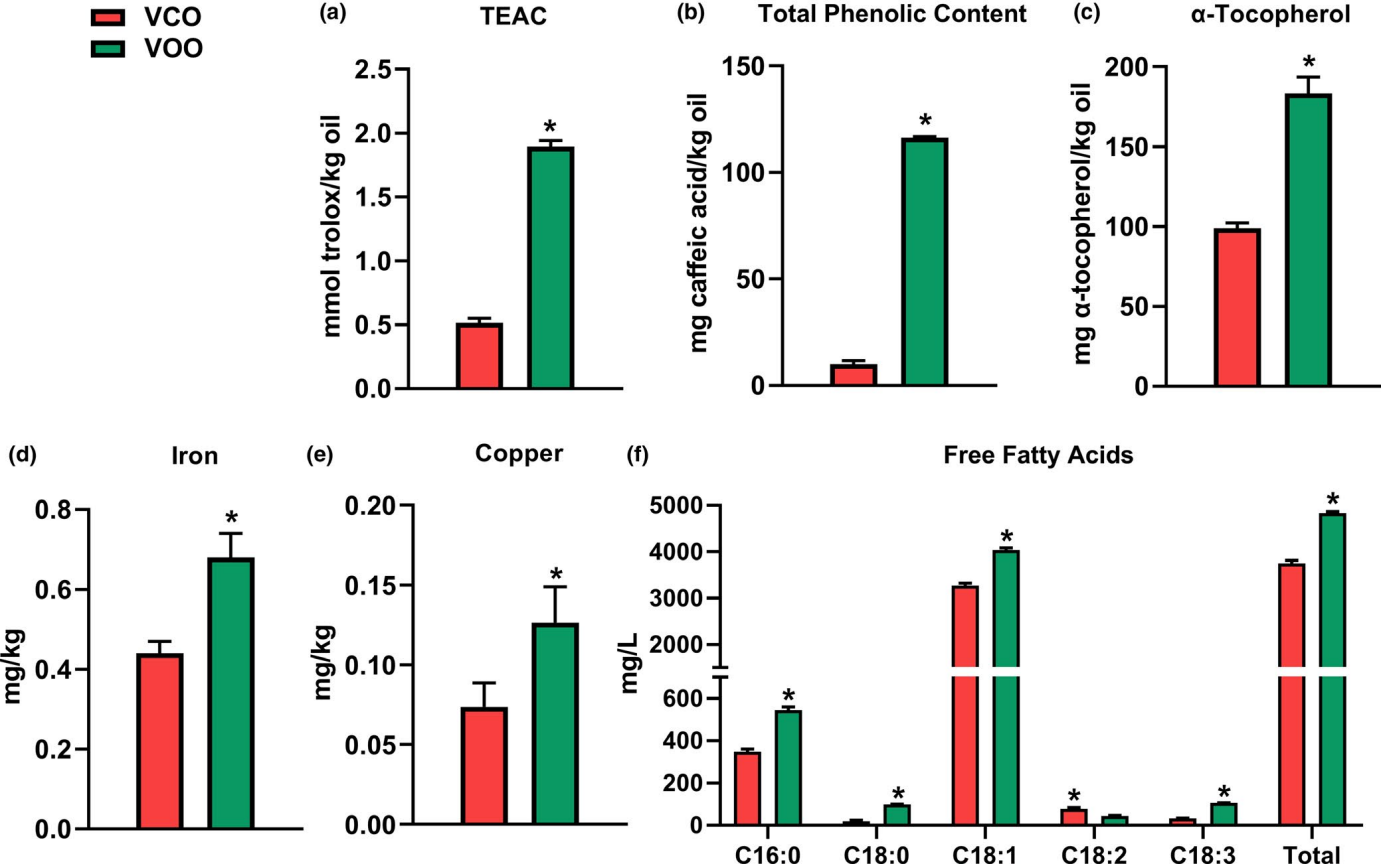
Antioxidant and pro‐oxidant components in virgin camellia oil (VCO) and virgin olive oil (VOO). (a) Trolox equivalent antioxidant capacity (TEAC). (b) Total phenolic content. (c) α‐Tocopherol. (d) Iron. (e) Copper. (f) Free fatty acids. Each parameter was analyzed in triplicate. *p <.05

The TEAC, which reflects the free radical‐scavenging ability, of VOO was more than three times greater than that of VCO (Figure 6a). The total phenolic content in VOO was more than 10 times higher than that in VCO (Figure 6b). The α‐tocopherol concentration of VOO was also greater than that of VCO (Figure 6c). Overall, VOO had more antioxidants than VCO in this study. As for pro‐oxidant components, the concentrations of iron and copper in VOO were around 1.55 and 1.72 times higher than those in VCO, respectively (Figure 6d–e). In addition, VOO had higher contents of total and individual free fatty acids than VCO, except linoleic acid (Figure 6f). The concentrations of free stearic acid and free α‐linolenic acid in VOO were about five and three times greater than that of VCO, respectively.

## 4 DISCUSSION

Chemometric comparisons of camellia oil with other common edible oils, especially olive oil, revealed the chemical attributes that distinguish camellia oil from other oils on its chemical composition and thermal stability. The significance and causes of these attributes are discussed as follows.

### 4.1 TAG and fatty acid profiles of camellia oil

Even though fatty acid composition analysis is the most commonly conducted analysis on edible oils, TAGs, not free fatty acids, are indeed the natural and dominant species in edible oils. The number of TAGs is far greater than the number of fatty acids in an edible oil. Therefore, TAG profile has clear advantages over fatty acid profile to represent the identity of an edible oil. TAG profile has been used extensively to characterize olive oil cultivars (Aranda et al., 2004; Galeano Diaz et al., 2005). The TAG profile of camellia oil has also been examined in previous studies, including the positional distribution of fatty acids in the TAG molecules (Wei et al., 2016, 2019). However, little is known about the differences between camellia oil and other edible oils, especially olive oil, in their TAG profiles. In this study, triolein (T1: OOO) was present as the most abundant TAG specie in camellia, olive, canola, safflower, and peanut oils, but not in corn, soybean, and grapeseed oils. This observation is consistent with the fact that camellia, olive, canola, safflower (from olive oil infusion or genetic modification), and peanut oils are high‐oleic oils. Camellia and olive oils differ from canola, safflower, and peanut oils in the 2nd most abundant TAG specie, which is T2 (POO) in camellia and olive oils, but T5 (OOL) in canola, safflower, and peanut oils. The comparison of camellia oil versus olive oil further showed that both VCO and RCO have much higher level of T13 (OOG), a minor TAG, than olive oil. Therefore, the profile of T1 (OOO), T2 (POO), and T13 (OOG) could distinguish camellia oils from other cooking oils and may serve as useful markers for defining the authenticity of commercial camellia oil.

Species, cultivars, maturation, and environment have been shown to affect the TAG and fatty acid composition of olive oil (Aranda et al., 2004; Li et al., 2019; Mailer et al., 2010). These associations have also been found in camellia oil. Species appear to affect the fatty acid composition of camellia oil since the camellia oils extracted from 12 *Camellia* species growing in 17 regions of Taiwan varied greatly in the levels of oleic acid, linoleic acid, palmitic acid, and stearic acid, which ranged 41.1%–89.0%, 3.0%–34.0%, 5.1%–25.4%, and 2.0%–4.5%, respectively (Su et al., 2014). In contrast, the influence of selective breeding for cultivars might be limited as the fatty acid profiles of 10 *Camellia oleifera* cultivars in mainland China were comparable (Yang et al., 2016). The maturity of camellia seeds also affects the fatty acid composition of TAGs since the increases of OOO and SOO, and the decreases of PPL and SSO were detected in camellia oils during the maturity period (Zarringhalami et al., 2011). As for the influence of geographic environment, a negative correlation between stearic acid in camellia oil and latitude has been observed and attributed to the need for cold resistance (Yao et al., 2011). In this study, the levels of fatty acids in the examined VCO and VOO (Table 2) are within their reported ranges (Ollivier et al., 2003; Yang et al., 2016). VCO is less saturated than VOO, but VCO also contains less α‐linolenic acid. This feature of fatty acid composition is expected to affect the thermal stability of camellia oil.

### 4.2 LOP profile of camellia oil under thermal stress

Among three measured conventional lipid oxidation indicators, PV reflects the primary LOPs while TBARS and AnV measure secondary LOPs. The three prominent observations from these measurements on the VCO and the VOO after heating are the lack of evident increases of PV and TBARS; the dramatic increase in AnV; and the higher basal levels of PV, TBARS, and AnV in the VOO than those in the VCO. The PV profiles of VCO and VOO in this study were similar to the reported changes in PV after heating olive oil under different frying processes (Cao et al., 2020; Karakaya & Şimşek, 2011). Interestingly, the rapid decreases of PV at 185°C occurred simultaneously with the rapid increases of AnV in both VCO and VOO, implying the association between the decomposition of hydroperoxides and the formation of aldehydes and other secondary LOPs. The lack of changes in the TBARS value could be attributed to the fatty acid composition of camellia and olive oils as well as the occurrence of nonspecific reactions in TBARS assay. Both camellia and olive oils are rather low in PUFAs, which are the precursors of malondialdehyde (MDA), a secondary LOP functioning as the main target of TBARS assay (Ma et al., 2019). In addition, VCO and VOO, as virgin oils, contain many chemical components that could react with TBARS reagent, including aldehydes, carbohydrates, amino acids, and nucleic acids (Almandós et al., 1986; Buttkus & Bose, 1972). Those nonspecific reactions might contribute to the higher basal level of TBARS in unheated VOO than that of the VCO in this study. The different basal levels of PV and AnV between examined VCO and VOO could be contributed by many factors, including the natural lipid oxidation in seeds, manufacturing, and storage conditions (Velasco & Dobarganes, 2002; Zhu et al., 2020). Overall, AnV serves as a better indicator than PV and TBARS to reflect the oxidative status of camellia and olive oils under thermal stress. This conclusion is further supported by our recent studies on evaluating the oxidized soybean oils under different thermal treatments (Wang, Csallany, et al., 2016; Yuan et al., 2020).

Chemometric profiling of aldehydes yielded additional explanation on the observed changes in AnV and useful information on the thermal stability of tested VCO and VOO. A prominent feature of aldehyde heatmap (Figure 5m) is that the clustering of aldehydes is largely based on their fatty acid precursors. Among the aldehydes that increased continuously at 185°C (cluster B in Figure 5m), 2‐undecenal (X), 2‐decenal (IX), and octanal (V) mainly originate from the hemolytic *β*‐scission of 8‐, 9‐, and 11‐hydroperoxides of oleic acid, respectively (Cao et al., 2020; Ho & Chen, 1994), while 2‐nonenal (VI) might from further decomposition of 2‐undecenal (Warner et al., 2001). Among the aldehydes that peaked within 2 hr of 185°C heating in VOO (cluster A in Figure 5m), 2,4‐decadienal (VIII) and 2‐octenal (IV) are the known breakdown products of linoleic acid and trilinolein (Choe & Min, 2006; Warner et al., 2001) while 2,4‐heptadienal (II) is the oxidation products of α‐linolenic acid (Guillén & Uriarte, 2012; Ho & Chen, 1994). Interestingly, nonanal (VII) has been shown as a degradation product from oleic acid (Cao et al., 2020; Guillén & Uriarte, 2012), but its formation pattern was more comparable to the aldehydes degraded from linoleic acid, such as 2,4‐decadienal (VIII) and 2‐octenal (IV), than the ones from oleic acid. The association between aldehyde formation and fatty acid composition is also supported by our study on the aldehyde formation in soybean oil (Wang, Csallany, et al., 2016). In quantity, oleic acid‐derived aldehydes, such as 2‐undecenal and 2‐decenal, are more abundant in heated high‐oleic VCO and VOO in this study, while linoleic acid‐derived aldehydes, including 2,4‐decadienal and 4‐hydroxynonenal (4‐HNE), are dominant in heated high‐linoleic soybean oil (Wang, Csallany, et al., 2016). This difference in aldehyde profile is expected to affect the toxicological properties and safety of thermally oxidized oils since the correlations between aldehydes and animal performance have been observed in our recent study (Yuan et al., 2020).

### 4.3 Factors contributing to thermal stability of camellia oil

Thermal stability is an important quality index that reflect the ability of oils to resist degradations, especially oxidation degradation, under thermal treatments during processing. Thermal stability of an oil is strongly associated with its TAG and fatty acid compositions as well as its antioxidant and pro‐oxidant contents. As two high‐oleic oils with similar fatty acid profiles, olive oil and camellia oil are high‐quality cooking oils with high thermal stability. Interestingly, the examined VCO in this study had lowers levels of LOPs and better thermal stability than the examined VOO, as evidenced by PV, AnV, and aldehyde contents before and after heating. This observation was different from a previous study, in which heated olive oil had similar aldehyde profile and lower AnV compared with heated camellia oil (Cao et al., 2020). This discrepancy is likely contributed by the sources of tested oils in these two studies and their chemical components, including fatty acid composition, antioxidant, and pro‐oxidant contents. In this study, the fatty acid composition may favor olive oil in thermal stability since the VOO contains more SFAs while the VCO contains more unsaturated fatty acids (UFAs). One exception is α‐linolenic acid, which is more abundant in the VOO in comparison with the VCO. Despite a minor fatty acid in either oils, α‐linolenic acid can have significant contribution to the LOP formation in frying oils (Choe & Min, 2007). For example, the soybean oil containing 0.8% of α‐linolenic acid had lower levels of total polar compounds than the soybean oil containing 2% of α‐linolenic acid when fried at 190°C for 5 hr (Warner & Gupta, 2003). Interestingly, the antioxidant content also favors the VOO for better thermal stability in this study because the VOO had higher levels of α‐tocopherol, phenolics, and TEAC than those in the VCO (Figure 6a–c). The levels of α‐tocopherol (99.03 mg/kg) and total phenolics (10.08 mg caffeic acid equivalent/kg) in the VCO are relatively low, but still within the reported ranges in camellia oil, which are 12.3–771 mg/kg for α‐tocopherol (Yuan et al., 2017; Zhang, Wang, et al., 2019) and 4.1–39.47 mg/kg for phenolics (Wang et al., 2017; Zhong et al., 2006). The causes behind these low values were not examined in this study, but could be attributed to cultivar, geographic location, harvesting, oil extraction process, and storage condition (Wang, Yang, et al., 2018; Zhang, Pan, et al., 2019). Overall, these observations on fatty acids and antioxidants in tested VOO and VCO indicated that the unsaturation level and antioxidant contents are not the only determinants of thermal stability. Instead, the pro‐oxidant contents, that is, transition metals and free fatty acids, serve as better predictors of thermal stability in this study since the VCO had lower levels of these pro‐oxidant contents than the VOO. The pro‐oxidant function of transition metals is mainly through decreasing the activation energy at the initiation step of lipid oxidation and accelerating the decomposition of hydroperoxides (Choe & Min, 2006). In addition, this pro‐oxidant function of transition metals could be strengthened by the phenolic compounds in virgin oils because the reactions between them can convert oxidized iron and copper (Fe^3+^ and Cu^2+^) to their reduced states (Fe^2+^ and Cu^+^), which can then readily react with hydroperoxides (Briante et al., 2003; Keceli & Gordon, 2002). Therefore, higher levels of transition metals and phenolic contents in the VOO could jointly contribute to its higher LOP levels in this study. Furthermore, the VOO had higher levels of free fatty acids than the VCO. Free fatty acids can negatively affect the stability of vegetable oils since the lack of steric hindrance makes free fatty acid more vulnerable to oxidation than esterified fatty acids (Frega et al., 1999). Free fatty acids could also facilitate the decomposition of hydroperoxides through the catalytic effect from their carboxyl groups (Miyashita & Takagi, 1986). Beside metals and free fatty acids, other components of virgin oils, such as chlorophyll and carotenoids, could also possess pro‐oxidant functions under appropriate circumstances (Park et al., 2013; Usuki et al., 1984). Further studies are required to define how the antioxidant and pro‐oxidant components jointly affect the thermal stability of camellia oil.

## 5 CONCLUSION

Chemometric analysis revealed both common features and subtle differences between camellia and olive oils in TAG and aldehyde profiles. Three TAG species, that is, OOO, POO, and OOG could be used jointly to distinguish camellia oil from other cooking oils, including olive oil. The oxidative status of camellia oil can be effectively monitored by AnV and major C9‐C11 aldehydes, but not by PV and TBARS. Despite the lower levels of α‐tocopherol and phenolics, the examined VCO had lower levels of α‐linolenic acid, free fatty acids, and transition metals, which could contribute to its better thermal stability than the examined VOO. Therefore, instead of a single factor, the balance among fatty acid unsaturation level, antioxidants, including α‐tocopherol and phenolics, and pro‐oxidants, including transition metals and free fatty acids, may determine the thermal stability of cooking oils. Future investigations on how these factors jointly affect the thermal stability may facilitate the development of approaches and practices to preserve and improve the quality and functions of camellia oil in production and food processing.

## CONFLICT OF INTEREST

The authors declare that they have no conflict of interest.

## ETHICAL APPROVAL

Ethics approval was not required for this research.

## Supporting information

Supplementary Material

## Data Availability

The data that support the findings of this study are available from the corresponding author upon reasonable request.
