# Supplementary material for: Fingerprinting triacylglycerols and aldehydes as identity and thermal stability indicators of camellia oil through chemometric comparison with olive oil
Source: Food Sci Nutr. 2021 Mar 6;9(5):2561–75. doi: 10.1002/fsn3.2209 (PMC8116833; doi:10.1002/fsn3.2209)
Supplement: Supplementary file 1 — Supplementary Material [file FSN3-9-2561-s001.docx]

**Supplementary data**

**Table S1.** Sources of chemicals and reagents in chemical analysis, LC-MS analysis, structural confirmation and quantification

| **Chemicals and reagents** | **Sources** |
| --- | --- |
| Acetone-*d_6_*_,_ *p*-Anisidine, Malonaldehyde bis (dimethyl acetal), Potassium persulfate | Acros Organics (Morris Plains, NJ) |
| Acetonitrile (LC-MS grade), Ammonium formate, Concentrated nitric acid, regular (68-70%), Concentrated nitric acid, trace metal grade (67-70%), Ferrous chloride, Formic acid (LC-MS grade), n-Hexane (HPLC grade), Methanol (HPLC grade), Water (LC-MS grade) | Fisher Scientific (Houston, TX) |
| Ammonium acetate | Riedel-de Haen (Seelze, Germany) |
| 2,2’-Azino-bis (3-ethylbenzothiazoline-6-sulfonic acid) diammonium salt (ABTS) | Amresco (Solon, OH) |
| n-Butanol, Caffeic acid, Certified standard solutions of iron and copper (1000 mg/L), Folin-Ciocalteu phenol reagent, Magnesium nitrate, Potassium phosphate dibasic, Potassium phosphate monobasic, Sodium carbonate, 2-Thiobarbituric acid, α-Tocopherol, Trichloroacetic acid | Sigma-Aldrich (St. Louis, MO) |
| 1,2-^13^C_2_-Palmitic acid | CDN Isotope (Pointe-Claire, Quebec, Canada) |
| 2,4-Decadienal, 2-Decenal, 2,4-Heptadienal, Heptenal, 2-Nonenal, 2-Octenal, 2-Undecenal | Bedoukian Research (Danbury, CT) |
| 2,2’-Dipyridyl disulfide (DPDS) | MP Biomedicals (Santa Ana, CA) |
| Ethanol (200 proof) | Pharmaco-AAPER (Brookfield, CT) |
| Fatty acid standards (C4-C22), Triolein, Tripentadecanoin | Nu-Chek Prep, Inc (Elysian, MN) |
| Ferric chloride, 2-Hydrazinoquinoline (HQ), Octanal, Triphenylphosphine (TPP), Xylenol orange sodium salt | Alfa Aesar (Ward Hill, MA) |
| Glacial acetic acid, Potassium hydroxide, | Avantor (Radnor, PA) |
| Hydrochloric acid | RICCA Chemical Company (Arlington, TX) |
| 6-Hydroxy-2,5,7,8-tetramethylchroman-2-carboxylic acid (trolox) | Cayman Chemical (Ann Arbor, MI) |
| Nonanal, Pentanal, 2,2,4-Trimethylpentane (isooctane) | TCI America (Portland, OR) |

**Figure S1.** Representative mass chromatogram of triacylglycerol (TAG) T2 (POO). P: palmitic acid; O: oleic acid.

**
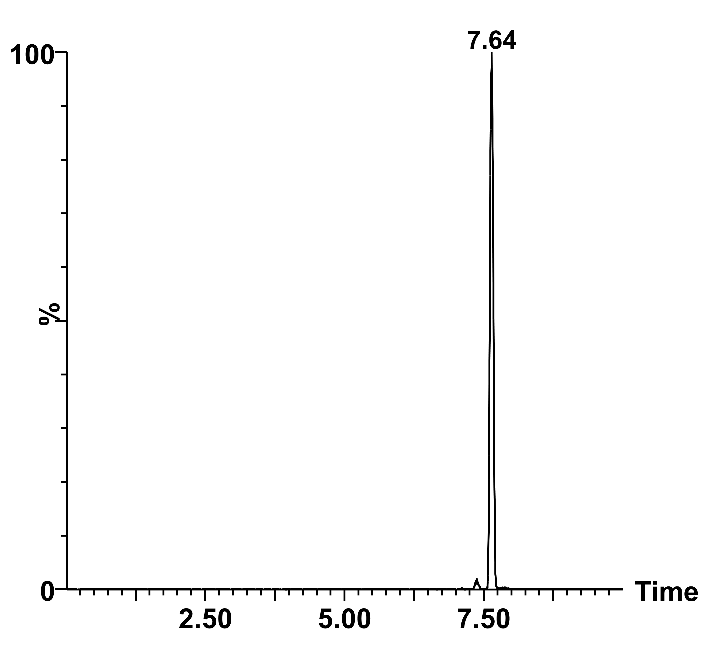
**

**Figure S2.** Preparation of heated virgin camellia oil (VCO) and virgin olive oil (VOO). (A) The temperatures and time points of sample collection. (B) Heat-induced color change in VCO. (C) Heat-induced color change in VOO. **
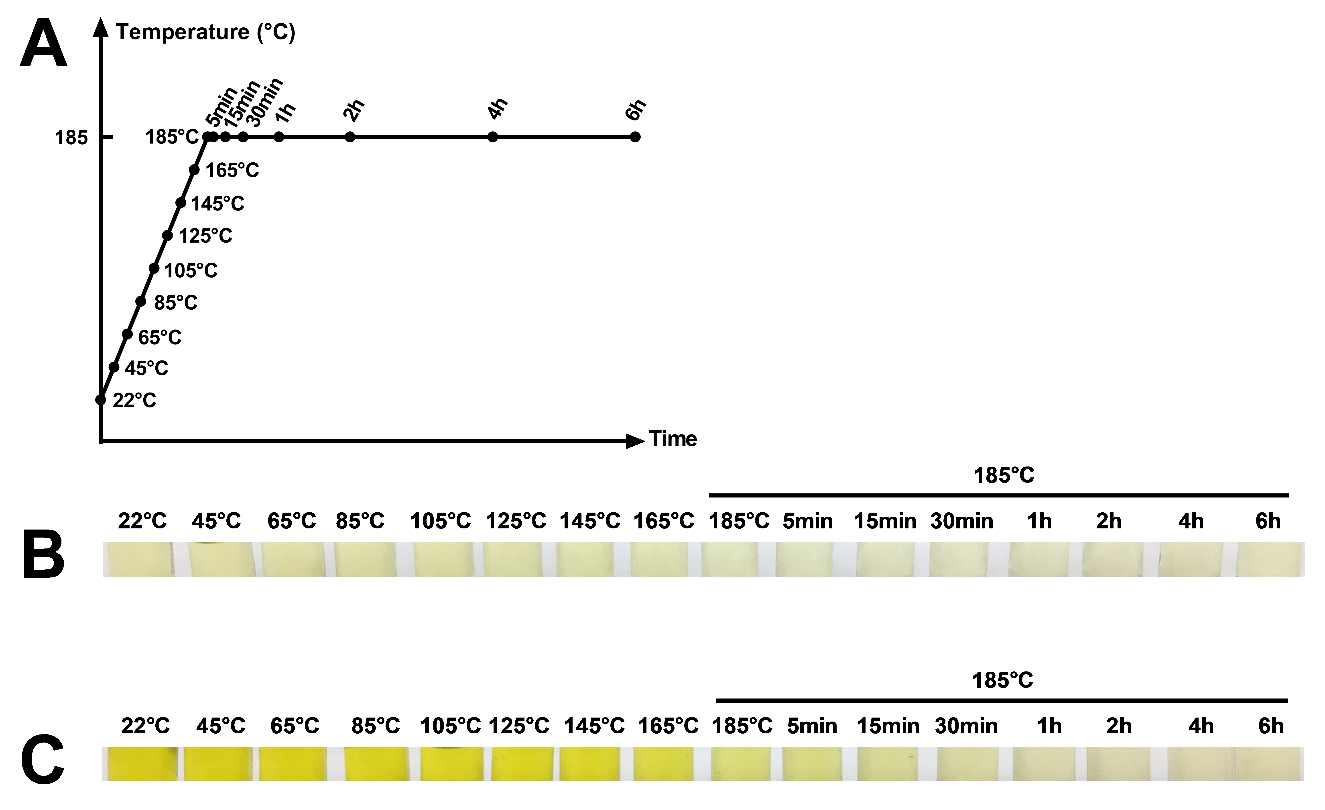
**
